# Nitrite Inhalants Use and HIV Infection among Men Who Have Sex with Men in China

**DOI:** 10.1155/2014/365261

**Published:** 2014-03-27

**Authors:** Dongliang Li, Xueying Yang, Zheng Zhang, Xiao Qi, Yuhua Ruan, Yujiang Jia, Stephen W. Pan, Dong Xiao, Z. Jennifer Huang, Fengji Luo, Yifei Hu

**Affiliations:** ^1^Chaoyang Center for Disease Control and Prevention, Beijing 100021, China; ^2^State Key Laboratory for Infectious Disease Prevention and Control, Beijing 102206, China; ^3^National Center for AIDS/STD Control and Prevention (NCAIDS), Chinese Center for Disease Control and Prevention (China CDC), Beijing 102206, China; ^4^Collaborative Innovation Center for Diagnosis and Treatment of Infectious Diseases, Beijing 102206, China; ^5^Department of Preventive Medicine, Vanderbilt University School of Medicine, Nashville, TN 37232, USA; ^6^School of Population and Public Health, University of British Columbia, Vancouver, British Columbia, Canada V6T 1Z4; ^7^Chaoyang Chinese AIDS Volunteer Group, Beijing 100021, China; ^8^Department of International Health, School of Nursing and Health Studies, Georgetown University, Washington, DC 20057, USA

## Abstract

*Objective*. This is the first study in China to examine the use of nitrite inhalants and its correlates among men who have sex with men (MSM) in Beijing, China. *Methods*. A cross-sectional survey was conducted in 2012. Structured interviews collected data on demographics, sexual and drug use behaviors, and the use of HIV services. Blood specimens were collected and tested for HIV and syphilis. *Results*. A total of 400 MSM eligible for the study were between 19 and 63 years of age and overall HIV prevalence was 6.0% (9.0% among nitrite inhalant users and 3.3% among nonusers). Nearly half (47.3%) of them reported ever using nitrite inhalants and 42.3% admitted using nitrite inhalants in the past year. Multivariable logistic analysis revealed that ever using nitrite inhalants in the past was independently associated with being aged ≤25 years, having higher education attainment, seeking sex via Internet, having casual partners in the past three months, and being HIV positive. *Conclusion*. The use of nitrite inhalants was alarmingly prevalent among MSM in Beijing. The independent association of the nitrite inhalant use with more casual sex partners and HIV infection underscored the need for intervention and prevention of nitrite inhalant use.

## 1. Introduction

Since 2007 sexual transmission has surpassed IDU and become the dominant mode of HIV transmission in China. The proportion of cumulative reported cases through homosexual transmission route has increased more than five times, from 2.5% to 13.7%, between 2006 and 2011 [1]. The national sentinel surveillance suggested that the HIV prevalence among men who have sex with men (MSM) increased from 0.9% in 2003 to 6.3% in 2011 [2]. It is estimated that homosexual transmission actually accounted for 29.4% of all newly infected HIV cases in 2011 [3]. Despite the implementation of numerous HIV prevention strategies targeting the promotion of safer sex [4, 5] and expanding needle exchange programs and methadone maintenance therapy [6], the HIV epidemic continues to expand among MSM.

Recent study from USA and Britain indicated that synthetic drug has increasingly become an important risk factor fueling the HIV epidemic [7–10]. However, limited data is available about synthetic drug use among MSM in China. Synthetic drugs such as amphetamine-type stimulants (ATS) and ketamine have become popular in entertainment industries and are confirmed to increase risk of HIV seroconversion [11, 12]. Studies also show that amyl nitrites appear to have the strongest association with HIV seroconversion among synthetic drugs [7, 13].

Since the 1960s, amyl nitrites have been popularly used as an inhalant among homosexual and bisexual men in order to relax the anal sphincter and diastolic capillaries [14, 15] and achieve enhanced sexual intercourse and euphoria [16–18]. Unfortunately, amyl nitrites also appear to pose multiple health risks that disproportionately impact MSM. Amyl nitrites use has been independently associated with unprotected anal intercourse [10] incident sexually transmitted infections, unprotected intercourse with serodiscordant partners, and HIV seroconversion among MSM [9, 19]. Commonly known as “poppers,” “rush,” or “rush poppers”, nitrite inhalants in China include isopropyl phenylenes nitrate (2-propyl nitrate) and isobutyl nitrite (2-methylimino-nitrate) [20] and have become increasingly popular in MSM community. Epidemiological research of nitrite inhalants and HIV risk is well documented in Western countries [19–25]; however, there is limited information from China. This is the first study in China to investigate the prevalence and correlates of the use of nitrite inhalants among MSM.

## 2. Materials and Methods

### 2.1. Study Settings

A cross-sectional study of nitrite inhalants use among HIV positive and HIV negative MSM was conducted using the baseline data from an ongoing prospective cohort study in Beijing. The participants of the cohort study were contacted every 6 months, received an HIV test, and completed a survey on sexual behaviors. The baseline study was conducted from July to October 2012 by researchers from Beijing Jingcheng Venereal Hospital. The outreach workers recruited MSM participants from the community using a mixed method including website advertisements, community outreach to MSM-frequented venues (e.g., MSM clubs, bars, and bathhouses), and peer referrals as well as recruiting clients of sexually transmitted disease (STD) clinics and voluntary counseling and testing (VCT) clinics. Inclusion criteria were biological male who (1) was at least 18 years old, (2) self-reported ever having sex with men during the past three months, (3) was willing to provide blood samples to test for HIV, (4) completed the questionnaire interview, and (5) was physically able and willing to provide written informed consent. Written informed consent was obtained prior to the interview, physical examination, and collection of blood samples.

### 2.2. Data Collection

Data were collected by self-administrated structured questionnaire based surveys with computer-assisted self-interviewing (CASI) technology. The information collected included sociodemographic characteristics (e.g., age, ethnicity, education, marital status, occupation, Beijing residency, income, and health insurance status), sexual behavior (e.g., self-reported sexual orientation, age of sexual debut, venue for seeking a male sex partner, lifetime number of regular and casual sexual partners, sexual behavior in the past 3 months, and condom use in receptive and insertive anal intercourse in the past 3 months), nitrite inhalant use (e.g., sources of nitrite inhalants, number of sexual partners while using nitrite inhalants, perceived effect of nitrite inhalants on number of sex partners, perceived effect of nitrate inhalants on sexual pleasure, duration of sexual activity, condom use, sexual role played when having anal sex and using nitrite inhalants, and frequency of nitrite inhalants use), history of ever having STD and HIV and syphilis infection status at present. The study protocol and informed consent procedures were reviewed and approved by the Ethics Review Committee of the Chaoyang Center for Disease Control and Prevention. Participants provided their written informed consent to participate in this study during the enrollment.

### 2.3. Specimen Collection and Laboratory Tests

The specimen collection and physical examination were performed by trained and experienced physicians at the Institute of STD/AIDS Prevention and Treatment, Beijing Jingcheng Venereal Hospital, Beijing, China. Each participant was assigned a unique code to link the anonymous questionnaire and blood specimen. HIV infection status was screened by an enzyme immunoassay (Wantai Biological Medicine Company, Beijing, China), and positive specimen were confirmed by HIV 1/2 Western blot assay (HIV Blot 2.2 WB; Genelabs Diagnostics, Singapore). Syphilis antibody was tested with the Treponema pallidum particle assay (TPPA, InTec Products, Inc., Xiamen, China).

### 2.4. Statistical Analysis

Questionnaire data collected by CASI were exported into a Microsoft Office Excel format and then merged with laboratory test results that had been independently entered and verified by two study staff members. Completed databases were then analyzed with Statistical Analysis System (SAS 9.3 for Windows; SAS Institute Inc., NC, USA) software. Descriptive analyses were performed to compare sociodemographic characteristics, sexual behaviors, and HIV infection status between nitrite inhalants users and nonusers. Pearson's chi-squared test was used to compare differences between nitrite inhalants users and nonusers for categorical variables, while *t* tests were used for the continuous variable of age. Logistic regression models were constructed to evaluate the associations between each variable with lifetime history of nitrite inhalants use and nitrite inhalants use in the past 3 months. Multivariable logistic regression was employed to identify significant predictors for lifetime history of nitrite inhalants use, nitrite inhalants use in the last 3 months, and nitrite inhalants use with unprotected anal sex. All variables that had *P* values < 0.1 in univariate analyses were simultaneously entered into the multivariable logistic model to reduce the risk for missing potentially relevant variables as well as reduce the impact of confounding variables. Crude odds ratios and adjusted odds ratios were calculated along with 95% confidence intervals (CI). Statistical significance was defined by *P* < 0.05.

## 3. Results

### 3.1. Demographics of Participants

A total of 611 MSM were approached to participate in this study. Of them, 74 participants declined to participate during the informed consent process and 137 participants did not satisfy the inclusion criteria. The final sample size was 400 participants enrolled in the study from July to October 2012. The average age was 30 years (SD = 7.1, a range from 19 to 63), two-thirds (68.2%) did not have official Beijing resident permits termed “*Hukou,”* 93.5% identified themselves as ethnically Han, 64.5% received college or higher level of education, 73.7% were unmarried, and 64.2% had monthly incomes above 3000 Yuan (equivalent to US$ himself 484, and comparable to the average income level in Beijing). Regarding sexual orientation, the proportion of participants identifying itself as homosexual and bisexual was 71.5% and 24.5%, respectively.

### 3.2. Sexual Behaviors

Of the participants, 72.0% reported ever seeking sex partners via the Internet (Table 1), 22.5% reported ever having unprotected anal sex in the last month, 26.8% reported ever having unprotected anal sex in the past 3 months, 65.3% reported having a casual sex partner in the past 3 months, 54.5% reported having multiple sex partners in past 3 months, and 45% reported that they intended to have protected anal sex with casual sex partners in the future 3 months and 44% with regular sex partners.

### 3.3. The Use of Nitrite Inhalants

Of the participants, 47.3% reported ever using nitrite inhalants and 41.8% used nitrite inhalants at least once in the past year. Most participants had only begun using nitrite inhalants recently; the median time between initial use of nitrite inhalants and participation in the survey was one year. Participants on average snorted nitrite inhalants about twice (2.1) during their first experience of nitrite inhalants riding sex. The median reported that duration of efficacy for nitrite inhalants per use was 2 minutes (average duration: 3.8 minutes). Of 400 participants, 40.5% reported having sex with only one person after nitrite inhalants use and 4.5% reported having ≥2 sexual partners. Overall, 37.8% reported no change in the number of sex partners since starting to use nitrite inhalants, 36.0% reported that nitrite inhalants increased sexual pleasure, and 76.1% reported that their weekly frequency of having sex did not change after starting to use nitrite inhalants (Table 1). Participants who used nitrite inhalants were younger (59.8% versus 44.0%, *P* = 0.009), had higher educational attainment, were more likely to be single, and sought sexual partners via the Internet.

### 3.4. HIV Infection, Syphilis, and Nitrite Inhalants

HIV prevalence was six percent overall (24/400) and was significantly higher among those who ever used nitrite inhalants (9.0%, 17/189) compared with those who had never used nitrite inhalants (3.3%, 7/211) (*P* = 0.017). Syphilis prevalence was 22.8% overall (91/400) and was not significantly correlated with nitrite inhalants usage, lifetime or recent.

### 3.5. Correlates for Ever Using Nitrite Inhalants in the Lifetime or in the Past 3 Months

Multivariable logistic analysis showed that ever using nitrite inhalants in the lifetime was associated with younger age (<25 years), higher level of educational attainment, seeking partners via the Internet, and HIV infection (Table 2). Nitrite inhalants use in the last 3 months was associated with higher level of educational attainment, seeking partners via the Internet, and having multiple partners (Table 2).

## 4. Discussion

To the best of our knowledge, this is the first study in China to investigate HIV infection and the use of nitrite inhalants among MSM. This study revealed that the use of nitrite inhalants became alarmingly prevalent among MSM in Beijing and that the use of nitrite inhalants is associated with HIV infection. Half of the participants (47.3%) ever used nitrite inhalants in the past and 42.3% used nitrite inhalants in the past year. The prevalence of the use of nitrite inhalants in the past year found in this study is higher than that reported from studies (13%–35%) in both Western countries and other Asian countries [20, 22, 26–32]. Only 5% reported starting to use nitrite inhalants more than one year ago. Moreover, because the median time interval since initial use of nitrite inhalants to participation in the survey was only one year; it appears that the use of nitrite inhalants is a relatively new phenomenon among Beijing MSM. Average age of initial nitrite inhalant users was about 29 years old, which is similar to the age found in a study in the U.S [33].

Our study showed that MSM who use nitrite inhalants were more likely to be younger and with higher education. This finding is consistent with Reisner et al. [8], Buchbinder et al. [27], Chen et al. [34], and Benotsch et al. [35]. The younger and better educated MSM may have a community that has higher technical capability and is familiar with Internet market for substances. On the other hand they were also reporting more casual sex partners [17, 18] which can also be the consequences of cyber connections. Studies on how MSM use online social network to access drug and sex is urgently needed to improve our understanding of the role of online social net in nitrite use and HIV risk behaviors.

On the other hand, this study did not show any significant differences in condom use between participants who did and did not use nitrite inhalants, regardless of whether or not having anal sex was with regular or casual partners. This result is different from other studies [20, 26] due to differences in sampling and survey methodology. Brewer sampled participants by random-digit telephone dialing, while the present study did not use probability sampling but social network based mixed methods. Also, Choi conducted face-to-face based questionnaire interviews, while we conducted interviews with CASI. Anecdotal evidence from this study suggests that CASI seems to have less social desirability bias than face-to-face based interviews. Therefore we believe our study is more reliable for reporting of sensitive behaviors like condom use. Another possible explanation is that the nitrite user in our study has higher education and income level and hence more knowledge about HIV and better access to condoms. A future qualitative in-depth interview study with some of the MSM may help explain the finding.

Our study may start a hypothesis that, in certain social network of MSM, the emerging synthetic drugs such as nitrite inhalants may not have any impact on condom use. The importance of noninjection drug use as a risk factor for HIV infection among MSM in Asia seems to be on the rise [27]. In China, methamphetamine and other drug uses among MSM have been reported with relatively low prevalence among MSM; however, nitrite inhalants use as an emerging sex stimulant became prevalent very recently. The popularity of nitrite over other drugs may be due to the fact that methamphetamines are illegal in China; whereas, nitrite inhalants are new and remain legal. There is a common perception that nitrite inhalants have no effect on risk behaviors, which facilitated their high prevalence among MSM. Certainly the fact that nitrite is legal and methamphetamine is illegal can generate reporting bias.

The present study also showed that odds of HIV infection among MSM who used nitrite inhalants were 3.1 times higher than nonusers, a finding that is consistent with other longitudinal and cross-sectional studies [7, 27, 36–39]. However, it is interesting that there was no correlation found between nitrite use and syphilis. One reason may be that new infection and long-term infection of syphilis were not differentiated, as the prevalence of syphilis included past infections that had already been treated as well as active infections. In addition, we asked about drug use by injection and none of the participants reported ever being injected with drug.

## 5. Findings to Informing Intervention

Additional analysis was conducted on intention to have protected anal sex in the future 3 months with regular partners and casual partners (data are not shown). Participants with younger age and higher education attainments and seeking sex partners via the Internet were more likely to be ready to consistently use condoms in the future 3 months. This could be useful information for policy makers and health professionals considering future intervention plans. Today, younger MSM are more likely engaging in modern social networks such as dynamic virtual lives via emerging media. Thus, Internet based social networks such as [40] “Weibo” (a microblog similar to “twitter” in China), QQ, “wechat,” texting message, and “Apps” via mobile phone/The Internet could be effective channels to deliver interventions on sexual stimulants and promote safer sex. Web-based social networks help overcome social isolation from migrant status and migrant young men were more likely to use them to locate new sexual partners. As two-thirds of our participants did not have Beijing residency permits, it is imperative to pay attention to migrant young men, despite the lack of significant correlation with nitrite inhalant use. Among recent nitrite inhalant users, safer sex was associated with having multiple casual sex partners, younger age, and seeking partners via Internet. Participants with more partners may have perceived a higher chance of encountering HIV infected individuals, as a recent Hong Kong study showed [41]. According to Theory of Planned Behavior (TPB) [42], behavioral intention strongly predicts actual behavior in the future [43] or it may affect the behavior directly [44]. Educational information about the risks of HIV transmission from multiple unprotected sex partners and nitrite inhalants use should be disseminated via the Internet [41].

There were several limitations to this study. First, the cross-sectional design of this study precluded our ability to determine causal relationships. Results only indicate association between HIV infection and the use of nitrite inhalants, but temporal precedence could not be determined. Second, due to the fact that MSM are a socially “hidden” population, participants were recruited by nonprobabilistic sampling. Hence results may not have been representative of MSM in Beijing. However, HIV prevalence in the participants in the present study (6.0%) was similar to the other studies (5.6–6.5%) in Beijing, which recruited participants via respondent driven sampling, as well as HIV prevalence among MSM nationwide (6.3%) [45]. Third, participant responses may have been conditioned by social desirability that may have led to underreporting or overreporting of risky behaviors. Fourth, participant responses may have been influenced by recall bias, particularly lifetime events that may have occurred several years ago. In addition, our study did not differentiate recent or past syphilis status, since TPPA testing can only determine if the individual was ever infected with* Treponema pallidum*. The prevalence of TPPA positivity is comparable to previous reports [46]. The comparable age of the participants in other surveys [47, 48] in Beijing is another reflection of the representativeness of our sample. In spite of these limitations, our findings have important implications for public health intervention. We believe that the surge in common use of nitrite inhalants could fuel the rapid spread of HIV transmission. This cross-sectional study will serve as an important data source about novel recreational drugs among MSM in China and will help guide policy makers in developing appropriate HIV prevention policies.

In summary, this study revealed that the use of nitrite inhalants is alarmingly prevalent among MSM in Beijing. This study showed that the use of nitrite inhalants was associated with HIV infection. The independent association of nitrite inhalant use with more casual sex partners and HIV infection underscored the need for intervention and prevention of nitrite inhalant use and unprotected sex among MSM in Beijing. Further research is needed to assess the impact of MSM social network in terms of substance use and sexual risk behaviors as well as nitrite inhalant use on the expanding HIV epidemic among MSM in China. HIV prevention strategies should incorporate the component of harm reduction addressing the use of nitrite inhalants.

## Figures and Tables

**Table 1:** Demographic and nitrite inhalants use characteristics of men who have sex with men (MSM) in Beijing, 2012 (*N* = 400).


Characteristics	*N*	%

Age		
Mean (SD)	30.0 (7.1)	
Median (*Q*1, *Q*3)	29 (25, 33)	
Range	19–63	
Age		
<**25 years**	82	20.5
≥**25 years**	318	79.5
Ethnicity		
Han	374	93.5
Other	26	6.5
Married		
Yes (married, cohabiting, or divorced)	105	26.3
No	295	73.7
Years of schooling		
≤12	142	35.5
>12 (college and above)	258	64.5
Residency permit		
Beijing	127	31.8
Other	273	68.2
Monthly income		
≤3000 (CNY, 485USD)*	143	35.8
>3000	257	64.2
Sexual orientation		
Homosexual	286	71.5
^1^Other	114	28.5
Ever used nitrite inhalants		
Yes	189	47.3
No	211	52.8
Venue for seeking a male sex partner		
Internet	288	72
Other (park, bathroom)	112	28
Nitrite inhalants use in P12M		
Yes	167	41.8
No	17	4.3
Frequency of sex when using nitrite inhalants		
Never	3	0.8
Rarely	56	14
Sometimes	15	3.8
Every time	115	28.8
Having unprotected anal sex in last month		
Yes	90	22.5
No	135	33.8
Having unprotected anal sex in P3M		
Yes	107	26.8
No	133	33.3
Having casual sex partner in P3M		
Yes	261	65.3
No	139	34.7

Characteristics	*N*	%

Sources of nitrite inhalants		
Internet	49	12.3
Friends	114	28.5
Other sources	26	6.5
Number of sexual partners while using nitrite inhalants		
<2	162	40.5
≥2	18	4.5
Number of change of sexual partners since starting to use nitrite inhalants		
Increased	14	3.5
Decreased	7	1.8
No change	151	37.8
Not sure	17	4.3
Sexual role played when having anal sex and using nitrite inhalants		
Exclusive/mainly insertive	89	22.3
Exclusive/mainly receptive	74	18.5
Both, about the same	26	6.5
Nitrite inhalant use increases sexual pleasure		
Yes	144	36.0
No	45	11.3
Duration of sexual activity when using nitrite inhalants		
Longer	53	13.3
Shorter	14	3.5
Same	99	24.8
Frequency of condom use since starting use of nitrite inhalants		
Never use	14	3.5
Sometimes use, more frequently than before	10	2.5
Sometimes use, less frequently than before	16	4.0
Almost the same	48	12.0
Consistent condom use	101	25.3
Having multiple sex partners		
Yes	218	54.5
No	145	36.3
Number of casual partners in P3M		
1	88	22.0
2–5	87	21.8
>5	14	3.5
Having intentional protected anal sex with casual sex partners in F3M		
Yes	180	45.0
No	220	55.0
Having intentional protected anal sex with regular sex partners in F3M		
Yes	176	44.0
No	224	56.0
HIV serostatus		
Positive	24	6.0
Negative	376	94.0
Syphilis serostatus		
Positive	91	22.8
Negative	309	77.2

Note: *exchange rate 6.15–6.2 : 1 (CNY to USD); ^1^other = heterosexual, or bisexual, or unknown; P12M: the past 12 months; P3M: the past 3 months. Numbers might not add to totals due to missing data; F3M: the future 3 months.

**Table 2:** Predictors for nitrite inhalants use among MSM in Beijing (*N* = 400).

Factors	*N* (% )	Crude odds ratio (95% CI)	Adjusted odds ratio (95% CI)
Model 1	Lifetime nitrite inhalants use		

Age			
≥**25**	140 (44.0)	0.53 (0.32, 0.87)*	0.47 (0.27, 0.83)^†^
<25	49 (59.8)		
Ever married			
No	152 (51.5)	1.95 (1.23, 3.10)^†^	
Yes (others)	37 (35.2)	1.00	
Years of schooling			
>12	140 (54.3)	2.25 (1.47, 3.44)^†^	2.02 (1.24, 3.29)^†^
≤12	49 (34.5)	1.00	1.00
Sexual orientation			
Homosexual	147 (51.4)	1.81 (1.16, 2.83)^†^	
Others	42 (36.8)	1.00	
Venue for seeking sex partners			
Internet	158 (54.9)	3.18 (1.98, 5.10)^†^	2.49 (1.45, 4.28)^†^
Others (park, bathroom)	31 (27.7)	1.00	1.00
Number of male sex partners in P3M			
** **>**1**	119 (54.6)	1.92 (1.29, 2.87)^†^	
≤**1**	61 (42.1)	1.00	
Protected anal sex in P3M			
Yes	80 (44.9)	0.69 (0.46, 1.05)	
No	100 (54.1)	1.00	
Having a casual partner in P3M			
Yes	135 (51.7)	1.69 (1.11, 2.56)*	2.04 (1.26, 3.30)^†^
No	54 (38.9)	1.00	1.00
HIV serostatus			
Positive	17 (70.8)	2.88 (1.17, 7.11)*	3.16 (1.16, 8.67)*
Negative	172 (45.7)	1.00	1.00

Model 2	Recent nitrite inhalants use in the P3M		

Age			
≥**25**	62 (19.5)	0.7 (0.4, 1.2)	
<**25**	21 (25.6)	1.00	
Years of schooling			
>12	65 (25.2)	2.3 (1.3, 4.1)^†^	
≤12	18 (12.7)	1.00	
Sexual orientation			
Homosexual	64 (22.6)	1.4 (0.8, 2.5)	
Other	19 (16.7)	1.00	
Venue for seeking sexual partners			
Internet	73 (25.4)	3.5 (1.7, 7.0)^†^	3.9 (1.7, 8.9)^†^
Others (park, bathroom)	10 (8.9)	1.00	1.00
Number of male sex partners in P3M			
** **>**1**	57 (26.2)	2.1 (1.3, 3.6)^†^	5.0 (1.7, 14.9)^†^
≤**1**	26 (14.3)	1.00	
Protected anal sex with casual partners in P3M			
** Yes**	25 (18.8)	0.5 (0.3, 0.9)*	0.5 (0.2, 0.9)*
** No**	35 (32.7)	1.00	1.00
HIV serostatus			
Positive	5 (20.8)	1.0 (0.4, 2.8)	
Negative	78 (20.7)	1.00	
Syphilis serostatus			
Positive	16 (17.6)	0.8 (0.4, 1.4)	
Negative	67 (21.7)	1.00	

Note: P3M: the past 3 months; P12M: the past 12 months; *P < 0.05; ^†^ *P* < 0.01.
